# Preexisting Heterogeneity of Inducible Nitric Oxide Synthase Expression Drives Differential Growth of Mycobacterium tuberculosis in Macrophages

**DOI:** 10.1128/mbio.02251-22

**Published:** 2022-09-19

**Authors:** Ophélie Rutschmann, Chiara Toniolo, John D. McKinney

**Affiliations:** a School of Life Sciences, Swiss Federal Institute of Technology in Lausanne, Lausanne, Switzerland; Washington University School of Medicine in St. Louis

**Keywords:** *Mycobacterium tuberculosis*, macrophages, single-cell, heterogeneity, inducible nitric oxide synthase (iNOS), gamma interferon (IFN-γ), time-lapse, microscopy, innate immunity

## Abstract

Mycobacterium tuberculosis infection is initiated by the inhalation and implantation of bacteria in the lung alveoli, where they are phagocytosed by macrophages. Even a single bacterium may be sufficient to initiate infection. Thereafter, the clinical outcome is highly variable between individuals, ranging from sterilization to active disease, for reasons that are not well understood. Here, we show that the rate of intracellular bacterial growth varies markedly between individual macrophages, and this heterogeneity is driven by cell-to-cell variation of inducible nitric oxide synthase (iNOS) activity. At the single-cell level, iNOS expression fluctuates over time, independent of infection or activation with gamma interferon. We conclude that chance encounters between individual bacteria and host cells randomly expressing different levels of an antibacterial gene can determine the outcome of single-cell infections, which may explain why some exposed individuals clear the bacteria while others develop progressive disease.

## INTRODUCTION

A Mycobacterium tuberculosis infection usually starts when airborne droplets containing one or more bacteria enter an individual’s lungs and interact with alveolar macrophages (1). From this initial encounter, outcomes range from sterilization to latent infection or active disease (2). These heterogeneous outcomes may be linked to differences in the evolution of granulomas, the characteristic multicellular structures that form around bacteria in the lungs (3–5). However, clinical studies have shown that some individuals who are heavily exposed to M. tuberculosis remain tuberculin skin test negative and presumably uninfected, suggesting that it is possible for the host to suppress or clear the bacteria during the earliest stages of infection (6, 7). Thus, different infection outcomes may depend not only on granuloma evolution at later stages of the disease, but also on differences in the outcomes of interactions between M. tuberculosis and host cells during the initial phases of the infection.

Several findings support this hypothesis. During the first few weeks of infection, macrophages control M. tuberculosis growth differently due to differences in their metabolism or expression of immune-related genes, such as the gene for inducible nitric oxide synthase (iNOS) and genes downstream of NF-κB (8–10). Heterogeneous control of intracellular M. tuberculosis growth is observed not only in macrophages of different lineages, but also in homogeneous populations of primary macrophages cultivated *in vitro* (11). However, those previous studies did not distinguish whether cell-to-cell differences in the control of intracellular M. tuberculosis depended on different adaptation of infected host cells to the bacteria (12) or on preexisting heterogeneity in the host cells (13). Answering this fundamental question is particularly relevant in the context of tuberculosis, because an infection can start with a single bacterium coming in contact with a single host macrophage (14, 15). Thus, preexisting phenotypic heterogeneity among host cells, or heterogeneous cellular responses to intracellular bacteria, could influence whether an infection progresses or, conversely, the host is able to control and possibly eliminate the pathogen.

In this study, we investigated how preexisting phenotypic diversity in a population of macrophages contributed to the control of intracellular M. tuberculosis. To explore the behavior of individual macrophages infected with fluorescently labeled M. tuberculosis, we used single-cell time-lapse microscopy, a technique that has been successfully used to assess infection dynamics at the single-cell level and to investigate the links between host cell death and bacterial growth rate (16, 17). We observed that bacteria within the same macrophage displayed more similar growth rates than bacteria in different macrophages, suggesting that some host cells are better than others at controlling M. tuberculosis infection. Using fluorescent reporter macrophages, we found that iNOS expression varied between individual macrophages and fluctuated over time. This preexisting heterogeneity of iNOS expression explains the differential control of intracellular M. tuberculosis growth even within a clonal population of macrophages. Our findings highlight the importance of considering preexisting phenotypic heterogeneity in host cells when studying the pathophysiology of an infectious disease, as these differences may determine the outcome of the initial encounter between host and pathogen.

## RESULTS

### Intracellular growth of M. tuberculosis and survival of infected host cells are heterogeneous at the single-cell level.

**FIG 1:**
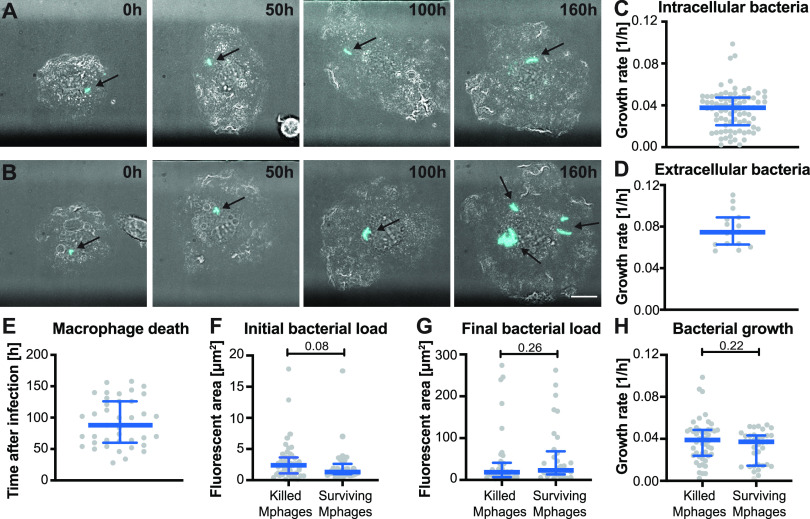
Intracellular growth of M. tuberculosis and survival of infected host cells are heterogeneous at the single-cell level. Murine BMDMs were infected with GFP-expressing M. tuberculosis and imaged by time-lapse microscopy at 2-h intervals for 168 h. (A) Example of an infected macrophage in which bacteria (cyan) grew slowly (doubling time, >168 h). (B) Example of an infected macrophage in which bacteria grew quickly (doubling time, <20 h). (C) Growth rates of intracellular M. tuberculosis. Each symbol represents a bacterial microcolony inside a single infected macrophage. (D) Growth rates of extracellular M. tuberculosis. Each symbol represents a single extracellular bacterial microcolony. (E) Survival time of infected macrophages that died before the end of the experiment. Each symbol represents a single macrophage. Survival time was calculated from the frame when initial infection occurred to the frame when death occurred. (F to H) Initial bacterial load (F), final bacterial load (G), and growth rate of intracellular bacteria (H) for macrophages that died during the experiment (killed Mphages) or macrophages that survived until the end of the experiment (surviving Mphages). Each symbol represents a single infected macrophage. Blue lines indicate median values and interquartile ranges. Scale bar, 10 μm. *P* values were calculated using a Mann-Whitney test.

We used fluorescence time-lapse microscopy to image individual mouse bone marrow-derived macrophages (BMDMs) infected with green fluorescent protein (GFP)-expressing Mycobacterium tuberculosis (Fig. 1A). The total fluorescent area per macrophage measured at 2-h intervals was used as a proxy for the number of intracellular bacteria and to calculate the growth rate of each intracellular microcolony. Macrophages can partially control M. tuberculosis growth, as the median growth rate of intracellular bacteria (0.036 per hour, corresponding to a doubling time of 27.8 h) (Fig. 1B) was reduced in comparison to extracellular bacteria growing on the debris of dead cells in the same culture (growth rate of 0.078 per hour, corresponding to a doubling time of 12.8 h) (Fig. 1C). Intracellular bacterial growth was heterogeneous: some intracellular bacteria grew with a growth rate above 0.08 per hour, corresponding to a doubling time of 12.5 h (Fig. 1A, lower panel, and Fig. 1B), while others grew very slowly, with doubling times of more than 1 week (Fig. 1A, upper panel, and Fig. 1B). Over 168 h of continuous imaging, ~60% of infected host cells died at different time points after infection (Fig. 1D). Surprisingly, the fate of individual infected macrophages (death or survival over the 168-h imaging period) did not correlate with the initial bacterial load (Fig. 1E), final bacterial load (Fig. 1F), or growth rate of intracellular M. tuberculosis (Fig. 1G; see also Fig. S1 in the supplemental material). Indeed, some macrophages survived even with a bacterial load that was higher than the median bacterial load in macrophages that died (Fig. 1F). It is worth noting, however, that even though no significant trend appeared, a subpopulation of exceptionally fast-growing intracellular bacteria with growth rates greater than 0.8 per hour, corresponding to a doubling time of less than 12.5 h, eventually did kill their host cells (Fig. 1G).

10.1128/mbio.02251-22.1 FIG S1 Identification of the time of death of individual macrophages in bright-field images. When macrophages die, they rapidly change shape (shrink), lose membrane integrity, and stop moving. Although it may be difficult to distinguish live and dead cells in individual snapshots, cell death events are easily identifiable by comparing adjacent frames in image series obtained by time-lapse microscopy. We defined the time of death for individual macrophages as the first image frame in which a cell was identified as dead (black arrow at 2 h). Scale bar, 10 μm. Download FIG S1, PDF file, 2.2 MB. Copyright © 2022 Rutschmann et al. 2022 Rutschmann et al. https://creativecommons.org/licenses/by/4.0/ This content is distributed under the terms of the Creative Commons Attribution 4.0 International license.

### Heterogeneity of intracellular M. tuberculosis growth rates is linked to variability in the macrophage population.

**FIG 2:**
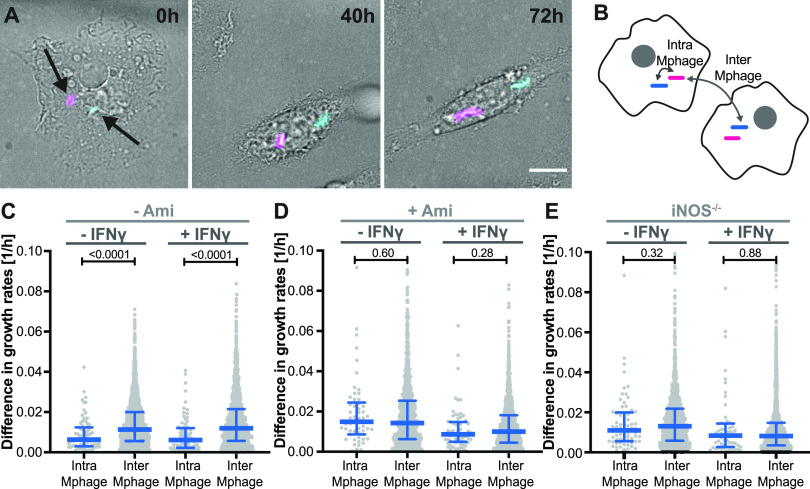
Single-cell variability of macrophage nitric oxide production drives heterogeneous growth of intracellular M. tuberculosis. Murine bone marrow-derived macrophages were simultaneously infected with M. tuberculosis strains expressing GFP or tdTomato and imaged by time-lapse microscopy at 1-h or 2-h intervals for 72 h. (A) Representative time-lapse images of a macrophage coinfected with GFP-expressing (cyan) and tdTomato-expressing (magenta) M. tuberculosis. (B) Schematic representation of the experimental design. The growth rates of two bacterial microcolonies (one green, one red) inside the same macrophage (intra-Mphage) or in two different macrophages (inter-Mphage) were compared. For calculation of intermacrophage differences in growth rate, each GFP-expressing bacterium was compared to all tdTomato-expressing bacteria not in the same macrophage. (C to E) Differences in growth rates between two bacterial microcolonies inside the same macrophage or in two different macrophages. Each symbol represents the difference in growth rates between one green and one red bacterium in unactivated or preactivated wild-type macrophages that were untreated (C), treated with aminoguanidine to inhibit nitric oxide production (D), or in untreated iNOS^−/−^ macrophages (E). Blue lines indicate median values and interquartile ranges. Scale bar, 10 μm. *P* values were calculated using a Mann-Whitney test.

The observed single-cell heterogeneity in intracellular M. tuberculosis growth rates could reflect heterogeneity in the bacterial population (some bacteria resist macrophage-imposed stresses better than others) or variability in the macrophage population (some cells control M. tuberculosis growth better than others). To address this question, we coinfected BMDMs with two fluorescently labeled M. tuberculosis strains expressing either constitutive GFP or tdTomato (Fig. 2A), which displayed similar intracellular growth rates (see Fig. S2). BMDMs infected with one bacterial cell of each color were imaged by time-lapse microscopy, and growth rates were calculated independently for intracellular bacterial microcolonies originating from a GFP^+^ or tdTomato^+^ bacterium. This approach permitted the comparison of two microcolonies (one GFP^+^, one tdTomato^+^) growing inside the same macrophage, or of two microcolonies growing in different macrophages (Fig. 2B). If variability in the macrophage population contributes to heterogeneity in intracellular bacterial growth rates, then two microcolonies in the same macrophage should behave more similarly than two microcolonies in different macrophages. We tested this hypothesis in unactivated macrophages and in macrophages preactivated with gamma interferon (IFN-γ), a cytokine that induces the expression of antibacterial host defense mechanisms (18) (see Fig. S2C). We found that the difference in growth rates between two intracellular bacterial microcolonies was smaller, on average, if they were in the same host cell than if they were in different cells, in both unactivated and IFN-γ-activated macrophages (Fig. 2C). These results suggest that some individual host cells control M. tuberculosis growth better than others, irrespective of their activation status.

10.1128/mbio.02251-22.2 FIG S2 Strains of M. tuberculosis expressing GFP or tdTomato exhibited similar intracellular growth rates in activated and unactivated macrophages. Murine bone marrow-derived macrophages (BMDMs) were simultaneously infected with M. tuberculosis strains expressing GFP or tdTomato and imaged by time-lapse microscopy at 1-h or 2-h intervals for 72 h. (A and B) Intracellular growth rates of M. tuberculosis in unactivated macrophages (*n* = 84 each for red and green bacteria) (A) and in IFN-γ-activated macrophages (*n* = 75 each for red and green bacteria) (B). Each symbol represents a bacterial microcolony growing inside a single macrophage. Blue lines indicate median values and interquartile ranges. *P* values were calculated using a Mann-Whitney test. (C) Growth rates of intracellular M. tuberculosis in unactivated (−IFN-γ) or preactivated (+IFN-γ) BMDMs, with or without aminoguanidine treatment (±Ami) to inhibit nitric oxide production. Each symbol represents a bacterial microcolony inside a single macrophage. Blue lines indicate median values and interquartile ranges. *P* values were calculated using a Mann-Whitney test. Download FIG S2, PDF file, 0.5 MB. Copyright © 2022 Rutschmann et al. 2022 Rutschmann et al. https://creativecommons.org/licenses/by/4.0/ This content is distributed under the terms of the Creative Commons Attribution 4.0 International license.

10.1128/mbio.02251-22.3 FIG S3 Aminoguanidine inhibits IFN-γ-induced production of reactive nitrogen species (RNS). Data shown are the concentrations of reactive nitrogen species (NO_2_ + NO_3_) in the supernatant of bone marrow-derived macrophages (A) or RAW 264.7 macrophages (B), with or without activation (IFN-γ) and with or without aminoguanidine (Ami) treatment to inhibit nitric oxide production. Error bars indicate standard deviations. *P* values were calculated using Student’s *t* test. Download FIG S3, PDF file, 0.4 MB. Copyright © 2022 Rutschmann et al. 2022 Rutschmann et al. https://creativecommons.org/licenses/by/4.0/ This content is distributed under the terms of the Creative Commons Attribution 4.0 International license.

### Single-cell variation in nitric oxide production by macrophages drives heterogeneous growth of intracellular M. tuberculosis.

Nitric oxide production by iNOS is one of the IFN-γ-induced mechanisms that macrophages use to control intracellular growth of M. tuberculosis (19, 20). We hypothesized that cell-to-cell differences in iNOS activity might explain why some host cells control M. tuberculosis growth better than others. We tested this hypothesis by coinfecting BMDMs with single GFP^+^ and tdTomato^+^ bacteria while inhibiting iNOS activity with aminoguanidine (Fig. 2C; see also Fig. S3A). In both unactivated and IFN-γ-activated BMDMs treated with aminoguanidine, intermacrophage and intramacrophage differences in bacterial growth rates were not significantly different (Fig. 2D). This suggests that, when iNOS activity is inhibited, all macrophages control M. tuberculosis growth about equally well. Consistent with this hypothesis, we found that intermacrophage and intramacrophage bacterial growth rates were not significantly different in BMDMs from iNOS^−/−^ mice. This observation held true in both unactivated and IFN-γ-activated iNOS^−/−^ BMDMs (Fig. 2E). Despite inhibition or lack of iNOS activity, IFN-γ activation was still effective in reducing intracellular bacterial growth (see Fig. S2C), presumably due to other IFN-γ-activated defenses, such as IRGM1 (21). We conclude that cell-to-cell variation in iNOS activity is linked to the control of intracellular M. tuberculosis growth in macrophages, irrespective of their activation status.

### Single-cell variability of macrophage iNOS expression in a reporter cell line contributes to heterogeneous growth of intracellular M. tuberculosis.

**FIG 3:**
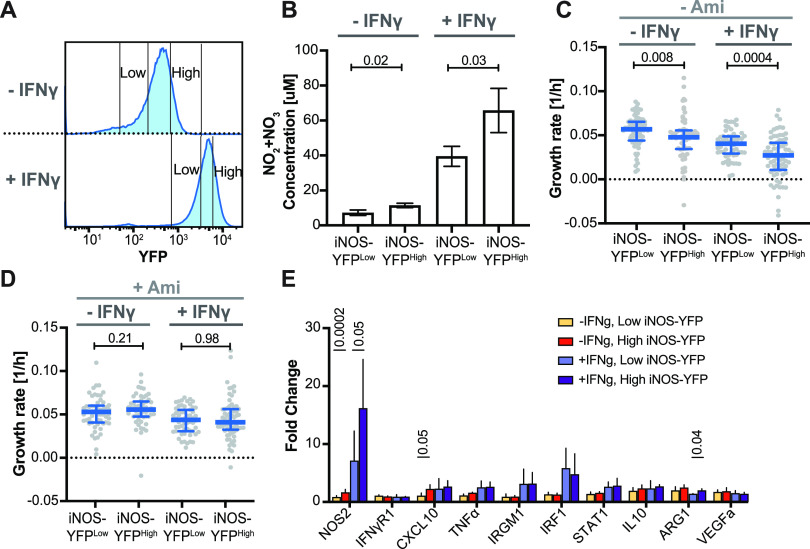
Single-cell variability of iNOS expression by macrophages contributes to heterogeneous growth rates of intracellular M. tuberculosis. Unactivated (−IFN-γ) and activated (+IFN-γ) RAW 264.7 macrophages that stably expressed YFP from the iNOS transcriptional promoter were flow-sorted into low- and high-fluorescence subpopulations prior to analysis. (A) Flow cytometry fluorescence profiles of iNOS-YFP-expressing macrophages. High-YFP and low-YFP gates are indicated. (B) The Griess assay was used to measure the cumulative concentration of reactive nitrogen species (NO_2_ + NO_3_) in the supernatants of macrophage subpopulations 24 h after sorting. *P* values were calculated using Student's *t* test. (C and D) Growth rates of intracellular M. tuberculosis in flow-sorted macrophage subpopulations were measured by time-lapse microscopy during 72 h. Macrophages were untreated (C) or treated with aminoguanidine to inhibit nitric oxide production (D). Each symbol represents a bacterial microcolony inside a single macrophage. Blue lines indicate median values and interquartile ranges. *P* values were calculated using a Mann-Whitney test. (E) Expression levels of selected genes involved in the IFN-γ response or linked to macrophage polarization in flow-sorted macrophage subpopulations. Relative expression (fold change) was normalized to an unactivated and unsorted sample. NOS2, IFN-γR1, CXCL10, IRGM1, IRF1, and STAT1 are involved in the IFN-γ response and, along with tumor necrosis factor α (TNF-α), are markers for M1 polarization. VEGFa and ARG1 are markers for M2 polarization. *P* values were calculated using Student's *t* test (*P* values of >0.05 are not shown).

To further investigate the link between cell-to-cell heterogeneity of iNOS gene expression and intracellular M. tuberculosis growth, we used RAW 264.7 reporter macrophages that expressed yellow fluorescent protein (YFP) from a copy of the iNOS promoter stably integrated in the genome (22). In these macrophages, iNOS-YFP is expressed at low basal levels in unactivated cells and is strongly induced upon activation with IFN-γ (Fig. 3A; see also Fig. S4).

10.1128/mbio.02251-22.4 FIG S4 Gating strategies for fluorescence-activated cell sorting of iNOS-YFP reporter macrophages. (A and B) Individual RAW 264.7 macrophages that stably expressed YFP from the iNOS transcriptional promoter were selected based on their forward scatter (FSC) and side scatter (SSC) (A) and by exclusion of doublets (B). (C and D) Live RAW 264.7 iNOS-YFP macrophages were selected as Sytox Red-negative (C) and YFP-positive (D) cells, which were subsequently used for flow sorting. (E) Gates were set to flow-sort iNOS-YFP^low^ and iNOS-YFP^high^ subpopulations comprising the 12% lowest-fluorescence and 12% highest-fluorescence cells in the distributions, respectively. Download FIG S4, PDF file, 0.5 MB. Copyright © 2022 Rutschmann et al. 2022 Rutschmann et al. https://creativecommons.org/licenses/by/4.0/ This content is distributed under the terms of the Creative Commons Attribution 4.0 International license.

To verify that iNOS-YFP expression was indeed linked to nitric oxide production, we flow-sorted the macrophages into low- and high-fluorescence subpopulations (Fig. 3A) and performed a Griess assay to measure the concentration of reactive nitrogen species (RNS) in the culture supernatants 24 h after sorting. iNOS-YFP^low^ cells produced less RNS than iNOS-YFP^high^ cells in both unactivated and IFN-γ-activated samples, confirming that iNOS-YFP expression is linked to RNS production (Fig. 3B).

We infected four flow-sorted subpopulations of macrophages (± IFN-γ iNOS-YFP^low^ and ± IFN-γ iNOS-YFP^high^) with tdTomato-expressing M. tuberculosis and used time-lapse microscopy to measure the growth rates of bacterial microcolonies within individual macrophages. In both unactivated and IFN-γ-activated macrophage subpopulations, iNOS-YFP^high^ cells controlled bacterial growth significantly better than iNOS-YFP^low^ cells (Fig. 3C). This difference was abolished when iNOS activity was inhibited with aminoguanidine, confirming that it is dependent on nitric oxide production (Fig. 3D; see also Fig. S3B). As we observed in infected BMDMs (see Fig. S2C), when iNOS activity was inhibited, IFN-γ-activated RAW 264.7 macrophages still controlled M. tuberculosis growth better than unactivated macrophages (Fig. 3D). For all conditions tested, the survival rate of infected macrophages was similar over the course of the experiments, indicating that the observed differences in bacterial growth rates were not due to differences in host cell viability (see Fig. S5).

10.1128/mbio.02251-22.5 FIG S5 Survival of M. tuberculosis-infected RAW 264.7 iNOS-YFP macrophages over time. Unactivated (−IFN-γ) and activated (+IFN-γ) RAW 264.7 macrophages that stably express YFP from the iNOS transcriptional promoter were flow-sorted into low- and high-fluorescence subpopulations and infected with M. tuberculosis. Individual infected macrophages were tracked using time-lapse microscopy and their time of death was quantified. Survival curves are shown for infected macrophages that were untreated (−Ami) (A) or treated with aminoguanidine (+Ami) (B). Under each condition, >70% of infected macrophages were still alive at the end of the experiment (after 72 h). Download FIG S5, PDF file, 0.4 MB. Copyright © 2022 Rutschmann et al. 2022 Rutschmann et al. https://creativecommons.org/licenses/by/4.0/ This content is distributed under the terms of the Creative Commons Attribution 4.0 International license.

### iNOS expression is not linked to differences in macrophage polarization or expression of other IFN-γ-regulated genes.

We investigated whether cell-to-cell differences in iNOS expression are linked to macrophage polarization or single-cell variability in IFN-γ-responsive gene expression by quantitative real-time PCR (qRT-PCR) analysis of unactivated or IFN-γ-activated iNOS-YFP^low^ and iNOS-YFP^high^ cells. These experiments confirmed that iNOS mRNA expression correlates with iNOS-YFP fluorescence levels (Fig. 3E). However, we did not observe any differences between iNOS-YFP^high^ and iNOS-YFP^low^ macrophages in the expression of other genes associated with macrophage polarization or the IFN-γ response (Fig. 3E).

### iNOS expression and RNS production fluctuate over time.

**FIG 4:**
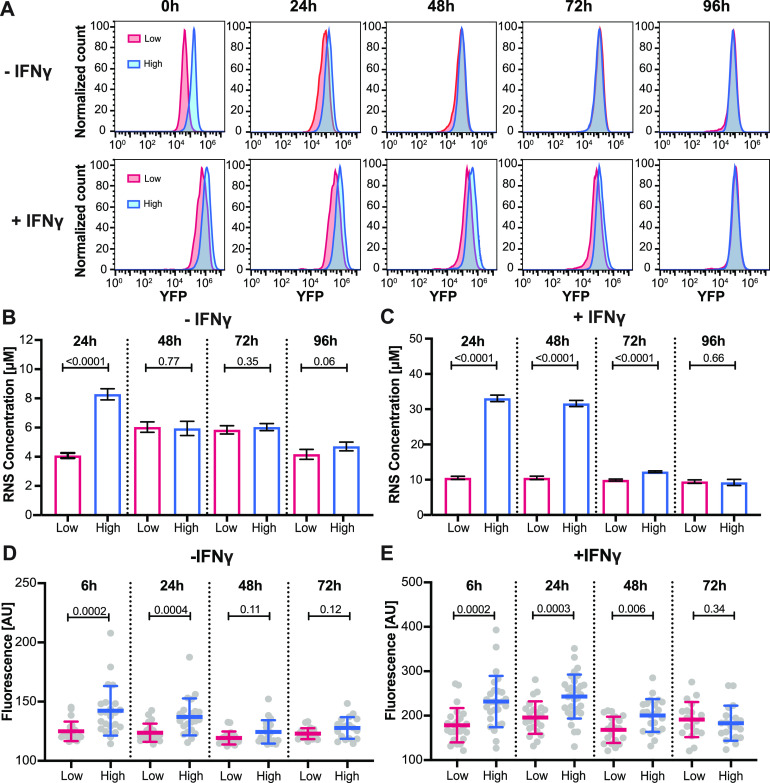
iNOS expression and RNS production fluctuate over time. Unactivated (−IFN-γ) and activated (+IFN-γ) RAW 264.7 macrophages that stably expressed YFP from the iNOS transcriptional promoter were flow-sorted into low- and high-fluorescence subpopulations prior to analysis. (A) Flow cytometry fluorescence profiles of iNOS-YFP-expressing macrophages 6, 24, 48, 72, and 96 h after sorting. (B and C) RNS concentrations in the supernatant of unactivated (B) and IFN-γ-activated (C) iNOS-YFP-expressing macrophages 24, 48, 72, and 96 h after sorting. *P* values were calculated using Student's *t* test. (D and E) Single infected macrophages were identified and tracked using time-lapse microscopy. Shown are the fluorescence of M. tuberculosis-infected unactivated (D) and IFN-γ-activated (E) iNOS-YFP-expressing macrophages 6, 24, 48, and 72 h after sorting as measured by microscopy. Each symbol represents a single infected macrophage. Blue lines indicate median values and interquartile ranges. *P* values were calculated using a Mann-Whitney test.

The observation that individual macrophages express iNOS-YFP at different levels prompted us to ask whether these cell-to-cell differences are stable or unstable over time. We used flow cytometry to measure fluorescence levels in flow-sorted macrophage subpopulations 0, 24, 48, 72, and 96 h after sorting. This analysis revealed that the iNOS-YFP^low^ and iNOS-YFP^high^ subpopulations were not stable over time and slowly converged toward each other in both unactivated and IFN-γ-activated samples (Fig. 4A). Convergence of the iNOS-YFP^low^ and iNOS-YFP^high^ subpopulations was faster in the unactivated samples than in the IFN-γ-activated samples. To confirm that the fluctuations in iNOS-YFP expression reflected changes in RNS production, we performed a Griess assay to measure levels of RNS secreted by iNOS-YFP^low^ and iNOS-YFP^high^ subpopulations 24, 48, 72, and 96 h after sorting. The amount of RNS secreted by the different subpopulations also fluctuated over time and converged within a similar time frame as iNOS-YFP expression for both unactivated and IFN-γ-activated samples (Fig. 4B and C).

Finally, to assess the impact of infection on the fluctuation of iNOS-YFP expression at the single-cell level, we infected flow-sorted subpopulations of macrophages with tdTomato-expressing M. tuberculosis and tracked single infected cells over 72 h using time-lapse fluorescence microscopy. As observed in our population-based experiments, we found that iNOS-YFP expression levels fluctuated in single cells and macrophages sorted into iNOS-YFP^low^ and iNOS-YFP^high^ populations converged over time and stabilized around the average level of gene expression found in the macrophage population prior to sorting (Fig. 4D and E; see also Fig. S6). These results confirmed that iNOS-YFP expression fluctuates independently of infection or IFN-γ activation, although activation may influence the time scale of these fluctuations.

10.1128/mbio.02251-22.6 FIG S6 Single-cell fluorescence traces of iNOS-YFP reporter macrophages. Unactivated (−IFN-γ) and activated (+IFN-γ) RAW 264.7 macrophages that stably express YFP from the iNOS transcriptional promoter were flow-sorted into low- and high-fluorescence subpopulations and infected with M. tuberculosis. Individual infected macrophages were tracked using time-lapse microscopy. Representative fluorescence profiles over time are shown for single unactivated (A) or activated (B) macrophages originating from the iNOS-YFP^high^ or the iNOS-YFP^low^ subpopulation. Download FIG S6, PDF file, 0.4 MB. Copyright © 2022 Rutschmann et al. 2022 Rutschmann et al. https://creativecommons.org/licenses/by/4.0/ This content is distributed under the terms of the Creative Commons Attribution 4.0 International license.

Despite the observed fluctuations in iNOS-YFP expression over time, we were able to measure significant differences in bacterial growth rates between iNOS-YFP^high^ and iNOS-YFP^low^ cells over the course of our experiments. These observations suggested that early exposure to different concentrations of intracellular RNS may be sufficient to drive differences in bacterial growth rates at early as well as late time points. We tested this hypothesis by comparing bacterial growth rates in unactivated iNOS-YFP^high^ and iNOS-YFP^low^ macrophages between 0 and 36 h and between 36 and 72 h. We found that intracellular bacterial growth rates were significantly different between iNOS-YFP^high^ and iNOS-YFP^low^ subpopulations during both the early and late time intervals (see Fig. S7), suggesting that differences in iNOS gene expression, even when limited to the early stages of infection, can have a long-lasting impact on bacterial growth rates.

10.1128/mbio.02251-22.7 FIG S7 Growth rates of intracellular M. tuberculosis in iNOS-YFP^low^ and iNOS-YFP^high^ macrophages. Unactivated (−IFN-γ) RAW 264.7 macrophages that stably express YFP from the iNOS transcriptional promoter were flow-sorted into low- and high-fluorescence subpopulations and infected with M. tuberculosis. The single-cell growth rates of intracellular bacteria were measured by time-lapse microscopy and quantified between 0 and 36 h (A) or 36 and 72 h (B). Blue lines indicate median values and interquartile ranges. *P* values were calculated using a Mann-Whitney test. Download FIG S7, PDF file, 0.4 MB. Copyright © 2022 Rutschmann et al. 2022 Rutschmann et al. https://creativecommons.org/licenses/by/4.0/ This content is distributed under the terms of the Creative Commons Attribution 4.0 International license.

## DISCUSSION

During the course of an infection, M. tuberculosis encounters heterogeneous niches ranging from different intracellular compartments to different types of cells and lesions (2, 23). This interplay between bacteria and heterogeneous host environments likely plays a role in determining the outcome of infection, ranging from disease progression to sterilization.

Here, we focused on the initial phases of an M. tuberculosis infection, during which small numbers of bacteria interact with individual host cells, to determine whether preexisting phenotypic heterogeneity in macrophages could drive differential growth of intracellular M. tuberculosis, potentially leading to different infection outcomes. We observed that individual cells can express different levels of iNOS within a clonal population of macrophages; this heterogeneity was not linked to differences in macrophage polarization, nor to the expression of other IFN-γ-related genes. Macrophages that expressed higher level of iNOS at the time of initial infection were more effective in controlling intracellular M. tuberculosis growth, suggesting that differences in expression levels of a single host antimicrobial gene could be sufficient to explain cell-to-cell variation in the control of intracellular bacteria. Our *in vitro* observations complemented a recent study showing that different populations of macrophages expressing different levels of iNOS coexist in the lungs of mice infected with M. tuberculosis and expression of iNOS correlates with expression of the bacterial stress marker HspX (10). Although the role of iNOS in protection against tuberculosis in humans remains controversial, expression of iNOS has recently been observed in lung sections from tuberculosis patients and in explanted human alveolar macrophages infected with M. tuberculosis (24, 25). Interestingly, human alveolar macrophages infected with M. tuberculosis show significant heterogeneity in the expression of several proinflammatory markers, including iNOS, which is correlated to their intracellular M. tuberculosis load (25). These studies suggest that heterogeneity in iNOS expression could have a role in disease progression *in vivo.*

Our observations that iNOS expression fluctuates in single macrophages and that flow-sorted iNOS-YFP^high^ and iNOS-YFP^low^ subpopulations converge toward an average level of iNOS expression over time (Fig. 4) are consistent with previous evidence that gene expression may be essentially stochastic at the single-cell level. According to this framework, genes are transcribed in bursts of variable intensity that occur at random time intervals (26–29). Based on these observations, we hypothesize that in individual macrophages iNOS expression might occur in bursts separated by silent intervals of variable duration. This model could explain our observation that flow-sorted subpopulations of iNOS-YFP^high^ and iNOS-YFP^low^ macrophages converged over time and eventually stabilized around the average level of iNOS expression found in the population prior to sorting. However, we cannot exclude that fluctuations in iNOS expression might reflect other mechanisms, such as cell cycle-dependent changes in gene expression or modifications in chromatin accessibility (30).

Cell-to-cell variation in iNOS expression seems to account for most of the intermacrophage heterogeneity in M. tuberculosis growth rates observed in our experiments. However, we also found that two bacteria growing inside the same host cell may exhibit different growth rates. Intramacrophage differences in M. tuberculosis growth rates could reflect occupancy of more or less permissive intracellular compartments by individual bacteria within the same macrophage (31–38). Alternatively, heterogeneous infection outcomes could also originate from the pathogen itself. Phenotypic heterogeneity in clonal bacterial populations is well documented and can be amplified by host stress, resulting in differences in bacterial fitness (12, 39–42). Cell-to-cell differences in the expression of bacterial virulence factors could also impact bacterial growth indirectly by driving different host-cell responses (43). It is thus likely that heterogeneous single-cell growth of intracellular bacteria is due to the interplay of different host and bacterial factors.

Our finding that preexisting heterogeneity in host cells can have an impact on the growth of intracellular M. tuberculosis is particularly relevant for tuberculosis, because even a single bacterium infecting a single permissive host cell may be sufficient to initiate an infection (14, 15). Macrophages have been reported to exhibit heterogeneity in the expression of many immune-related genes due to factors such as circadian rhythm, environmental variation, or age of the host, which could all impact how they respond to an initial infection (44–47). Our results suggest that differences in the expression of even a single antimicrobial gene, such as the gene for iNOS, could be sufficient to influence the outcome of infection. This conclusion could potentially be extended to any disease where the number of interacting host cells and bacteria is small at some stage of the infection (48). In such cases, chance encounters of the pathogen with host cells expressing different levels of antibacterial defense mechanisms could determine whether infection is controlled or progresses to active disease.

## MATERIALS AND METHODS

### Bone marrow-derived macrophages.

BMDMs were differentiated from frozen bone marrow stocks extracted from femurs of wild-type C57BL/6 mice or iNOS^−/−^ mice (B6.129P2-Nos2tm1Lau/J mice from Jackson Laboratories, catalog number 002609). The bone marrow was cultured in petri dishes in BMDM differentiation medium (Dulbecco’s modified Eagle’s medium [DMEM] with 10% fetal bovine serum [FBS], 1% sodium pyruvate, 1% GlutaMax, and 20% L929 cell-conditioned medium [as a source of granulocyte-macrophage colony-stimulating factor]) for 7 days. The adherent cells were then gently lifted from the plate using a cell scraper and resuspended in BMDM culture medium (DMEM with 5% FBS, 1% sodium pyruvate, 1% GlutaMax, and 5% L929 cell-conditioned medium). The macrophages were then seeded in 35-mm ibidi μ-dishes or in 4-compartment ibidi μ-dishes and allowed to adhere for 4 h at 37°C, 5% CO_2_ before use.

### RAW 264.7 iNOS-YFP macrophage cell line.

RAW 264.7 macrophages stably expressing YFP from a copy of the iNOS transcriptional promoter (22) were cultured in DMEM with 10% FBS, 1% Glutamax, and 1% sodium pyruvate at 37°C, 5% CO_2_. The macrophages were passaged every 3 days at a 1:4 ratio by gently lifting them off the culture flask with a cell scraper.

### M. tuberculosis strains.

GFP- and tdTomato-expressing M. tuberculosis Erdman strains were inoculated from frozen glycerol stocks in Middlebrook 7H9 (Difco) supplemented with 10% albumin-dextrose-saline (ADS), 0.5% glycerol, and 0.02% tyloxapol and cultured at 37°C with shaking.

### Flow sorting of RAW 264.7 iNOS-YFP reporter macrophages.

RAW 264.7 iNOS-YFP macrophages were detached from culture flasks with 10 mM EDTA. When required, macrophages were activated 24 h before detaching with 100 U/mL IFN-γ. The cells were then collected by centrifugation, resuspended in fluorescence-activated cell sorting (FACS) buffer (phosphate-buffered saline [PBS] with 1 mM EDTA), and sorted using a FACSaria Fusion system (the gating strategy is shown in Fig. S3 in the supplemental material). Sorted cells were collected, resuspended in DMEM with 10% FBS, 1% Glutamax, and 1% sodium pyruvate, seeded in μ-dishes, and allowed to adhere for at least 4 h before use.

### Macrophage infections.

For infection, 1 mL of M. tuberculosis culture at an optical density at 600 nm (OD_600_) of 0.5 was pelleted and resuspended in 200 μL of macrophage medium. Bacteria were passed through a 5-μm filter to eliminate aggregates. The resulting single-cell suspension was used to infect BMDMs or RAW 264.7 iNOS-YFP macrophages at a multiplicity of infection (MOI) of 1:1. When two fluorescent strains of M. tuberculosis were used simultaneously, both were added at an MOI of 1:1. After 4 h of infection, macrophages were washed extensively with macrophage medium to remove extracellular bacteria. When required, 100 U/mL IFN-γ was added to the macrophage medium 24 h before infection and maintained during the duration of the experiment. When required, 500 μM aminoguanidine was added to the culture medium at the time of infection and maintained thereafter.

### Time-lapse microscopy of macrophages infected with M. tuberculosis.

Infected BMDMs and RAW 264.7 iNOS-YFP macrophages were imaged with a DeltaVision microscope and a Nikon Ti2 microscope, respectively. A stage-top incubator (Okolab) was used to maintain the cells at 37°C in a humidified environment. Air mixed to 5% CO_2_ was supplied using an Okolab gas mixer. Infected BMDMs were randomly selected and imaged for up to 168 h. Macrophage medium was refreshed every 3 days via custom tubing connected to the lid of the ibidi μ-dish. Infected BMDMs were imaged using a 60× oil-immersion objective at 2-h intervals; 3 × 1 μm *z*-stacks were acquired for each point. Bacteria were identified by fluorescence emission on the green (GFP) or red (tdTomato) channel using fluorescein isothiocyanate (excitation [Ex] 490/20, emission [Em] 525/36) and tetramethyl rhodamine isocyanate (Ex 555/25, Em 605/52) filters, respectively. Infected RAW 264.7 iNOS-YFP macrophages were imaged for 72 h using a 40× air objective at 1-h intervals; 3 × 1 μm or 5 × 1 μm *z*-stacks were acquired for each point. iNOS-YFP levels were quantified and tdTomato-expressing bacteria were imaged using GFP (Em 480/30, Ex 535/45) and mCherry (Em 560/40, Ex 635/60) dichroic filters, respectively. For both BMDMs and RAW 264.7 iNOS-YFP macrophages, at least 25 infected cells were imaged per condition.

### Quantification of reactive nitrogen species (RNS) in cell culture medium.

BMDMs and RAW 264.7 iNOS-YFP macrophages were seeded in triplicates in 96-well plates with 100 μL of their respective culture medium at a concentration of 10^6^ cells/mL. When required, 100 U/mL IFN-γ or 500 μM aminoguanidine was added to the culture medium. After 24 h of incubation at 37°C, 5% CO_2_, 80 μL of culture supernatant was collected and centrifuged at 10,000 × *g* for 10 min. The concentration of RNS in the supernatant was measured using a nitrate/nitrite colorimetric assay kit (Abnova), as described by the manufacturer. Since RAW 264.7 macrophages divide approximately every 24 h, a different seeding strategy was used for time course experiments with these cells. Each population of flow-sorted RAW 264.7 iNOS-YFP macrophages was seeded in 4 wells of a 96-well plate at four different concentrations (10^6^, 0.5 × 10^6^, 0.25 × 10^6^, or 0.125 × 10^6^ cells/mL, all in 100 μL of medium) and measured at 24, 48, 72, and 96 h after sorting. This seeding strategy ensured that the samples used for different time points reached approximately the same number of cells. The plates were incubated at 37°C, 5% CO_2_ between the different time points. When required, 100 U/mL of IFN-γ was added to the medium of the cells directly after sorting.

### Quantitative real-time PCR.

Unactivated or preactivated (+100 U/mL IFN-γ) RAW 264.7 iNOS-YFP macrophages were sorted as described above. Directly after sorting, the macrophages were collected by centrifugation and lysed, and RNA was extracted using a Qiagen RNeasy micro kit plus according to the manufacturer’s instructions. DNase treatment was performed directly on the column during RNA extraction. The RNA was then reverse-transcribed with random hexamers using the SuperScript IV first-strand synthesis system (ThermoFisher). qRT-PCR mixtures were prepared using the SYBRGreen PCR master mix (Applied Biosystems) with 1 μM primers and 2 μL of cDNA. Reactions were run on an ABI Prism 7900HT sequence detection system (Applied Biosystems). Amplicon specificity was confirmed by melting curve analysis. Primer sequences were obtained from Origene. Primers were synthesized by Microsynth, Switzerland (see Table S1).

10.1128/mbio.02251-22.1 TABLE S1 Primer list. Download Table S1, DOCX file, 0.01 MB. Copyright © 2022 Rutschmann et al. 2022 Rutschmann et al. https://creativecommons.org/licenses/by/4.0/ This content is distributed under the terms of the Creative Commons Attribution 4.0 International license.

### Flow cytometry time course.

FACS-sorted unactivated or preactivated (+100 U/mL IFN-γ) RAW 264.7 iNOS-YFP macrophage subpopulations were seeded following the same seeding strategy described for the time course experiments to quantify RNS. For each subpopulation, 5 wells of a 24-well plate were seeded with 350 μL of cells at 2 × 10^6^, 10^6^, 0.5 × 10^6^, 0.25 × 10^6^, or 0.125 × 10^6^ cells/mL and analyzed by flow cytometry at 4, 24, 48, 72, and 96 h after sorting. The plates were incubated at 37°C, 5% CO_2_ between the different time points. When required, 100 U/mL IFN-γ was added to the medium of the cells directly after sorting. For analysis by flow cytometry, the cells were detached using trypsin, collected by centrifugation, resuspended in PBS plus 1 mM EDTA, and analyzed using a BD Accuri C6 flow cytometer.

### Image analysis.

Image analysis was performed using the FIJI version of the ImageJ software (49). All infected macrophages that survived for at least 24 h of imaging were analyzed. If a macrophage divided during the experiment, the daughter cell containing the bacteria was selected for continued analysis. If the bacterial microcolony was split between the two daughter cells, the analysis was stopped at this time point. All of the macrophages were imaged until the end of the experiment or until their death. The *z*-stacks acquired were projected into one image using a maximum intensity projection. A background subtraction was performed by subtracting from the fluorescence images a copy of the same images on which a Gaussian blur of 100-μm radius had been applied. Regions of interest corresponding to individual macrophages were manually drawn onto the phase images and transferred to the fluorescence images. A manual threshold was set on the fluorescence channel to segment the bacteria. The area above the threshold inside single macrophages was measured and used as a proxy for the number of intracellular bacteria for each time point. To quantify the growth rate of the intracellular bacteria, an exponential curve was fitted to the data. A similar method was used to measure the growth rate of bacteria identified as extracellular in BMDM infection experiments. Similarly, iNOS-YFP expression levels were quantified for each frame by transferring the manually drawn regions of interest corresponding to individual infected macrophages to the GFP fluorescence images. The average fluorescence intensity was measured for each individual macrophage and used as a proxy for iNOS-YFP expression levels. Cell death was identified using bright-field images. When macrophages die, they change shape (shrink), lose membrane integrity, and stop moving. Death events were identified by examining the subsequent time-lapse images for each cell (see Fig. S1). The time of death was manually annotated as the first time point at which death was observed. Out-of-focus images were manually excluded from analysis.

### Resource availability.

Further information and requests for resources and reagents should be directed to and will be fulfilled by the corresponding author, Chiara Toniolo.

### Materials availability.

This study did not generate new unique reagents.

### Data and code availability.

Any additional information required to reanalyze the data reported in this paper are available from the corresponding author upon request.
