# Supplementary figures and images for: Preexisting Heterogeneity of Inducible Nitric Oxide Synthase Expression Drives Differential Growth of Mycobacterium tuberculosis in Macrophages
Source: mBio. 2022 Sep 19;13(5):e02251-22. doi: 10.1128/mbio.02251-22 (PMC9600446; doi:10.1128/mbio.02251-22)

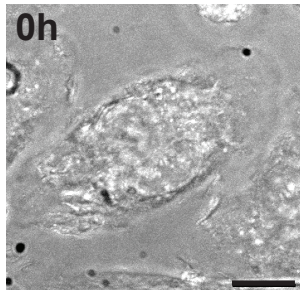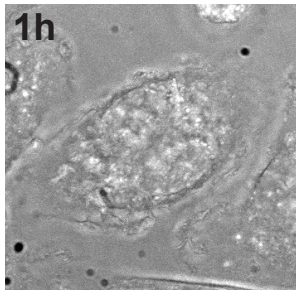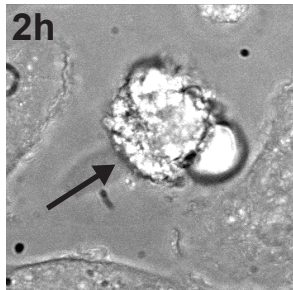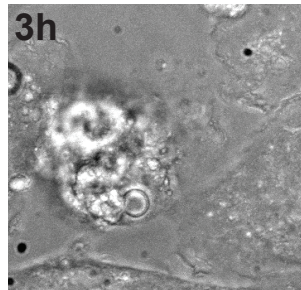

Supplement: FIG S1 [file mbio.02251-22-s0001.pdf]

**A**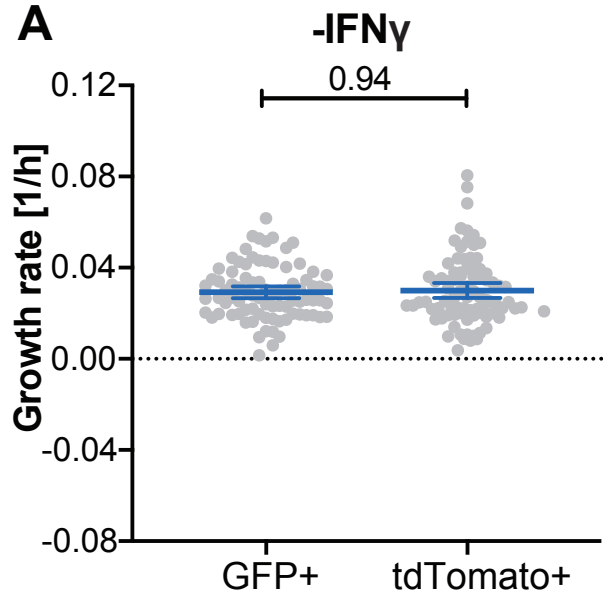**B**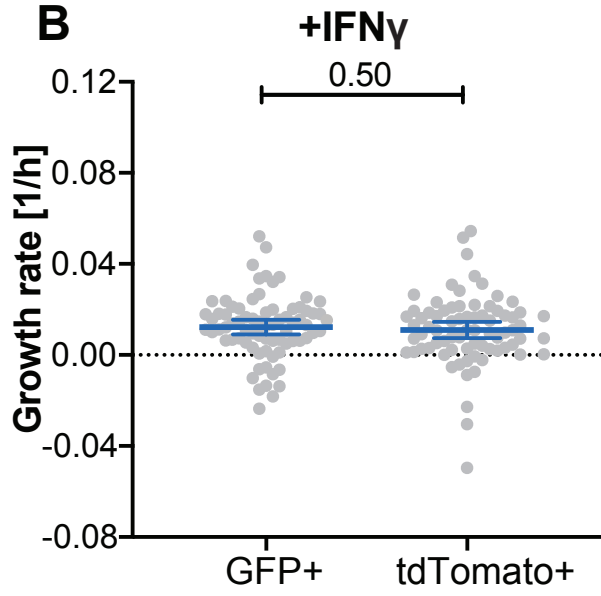**C**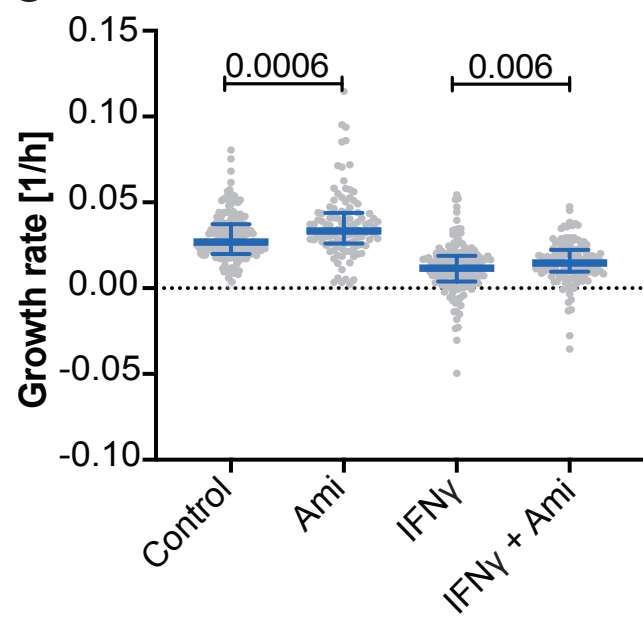

Supplement: FIG S2 [file mbio.02251-22-s0002.pdf]

**A****BMDMs**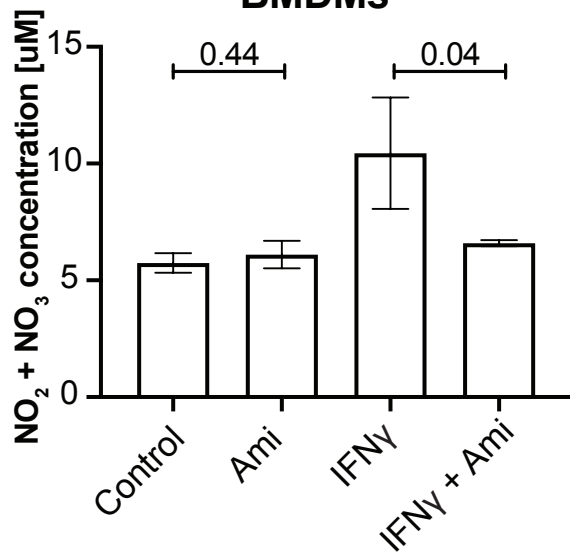**B****RAW Macrophages**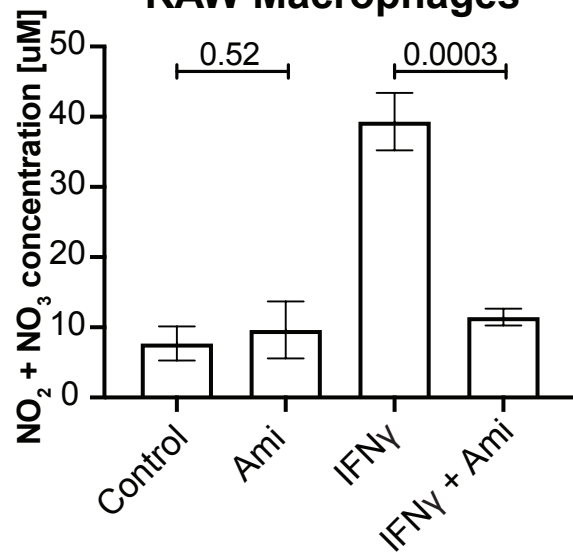

Supplement: FIG S3 [file mbio.02251-22-s0003.pdf]

**A**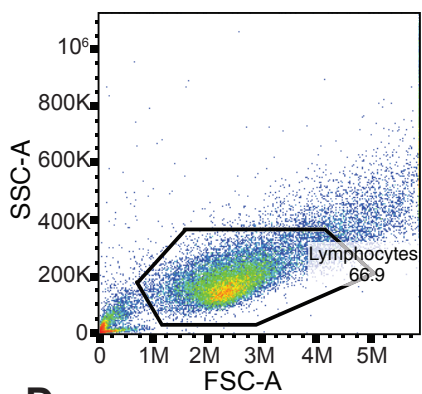**B**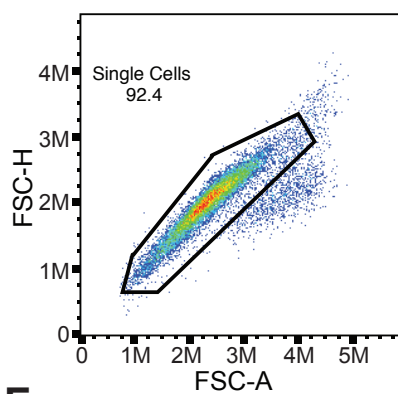**C**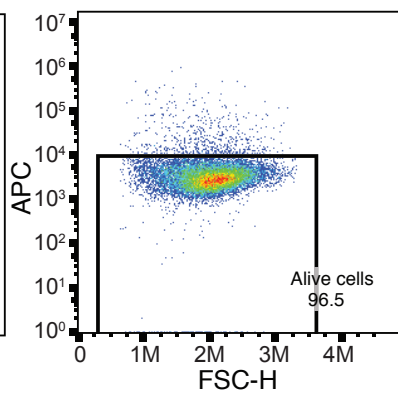**D**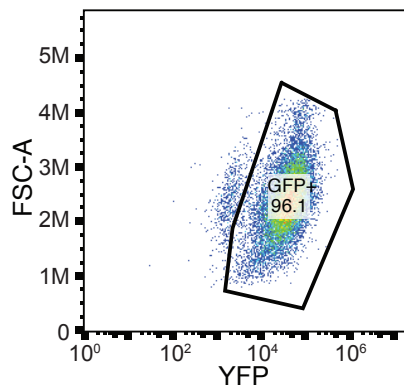**E**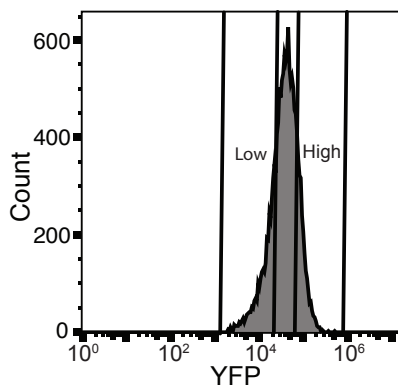

Supplement: FIG S4 [file mbio.02251-22-s0004.pdf]

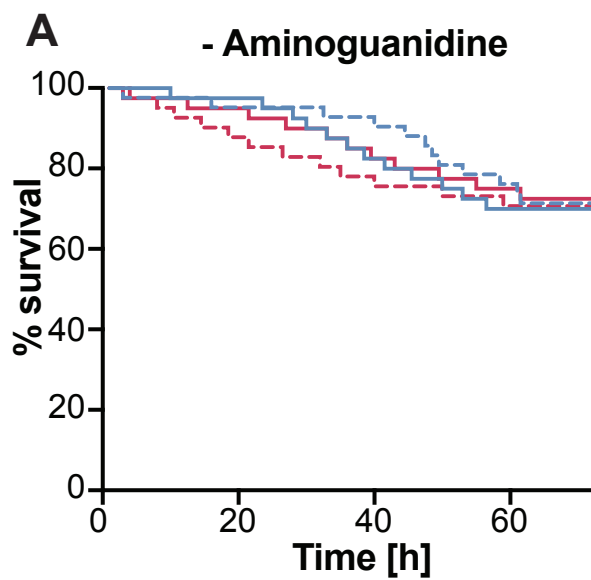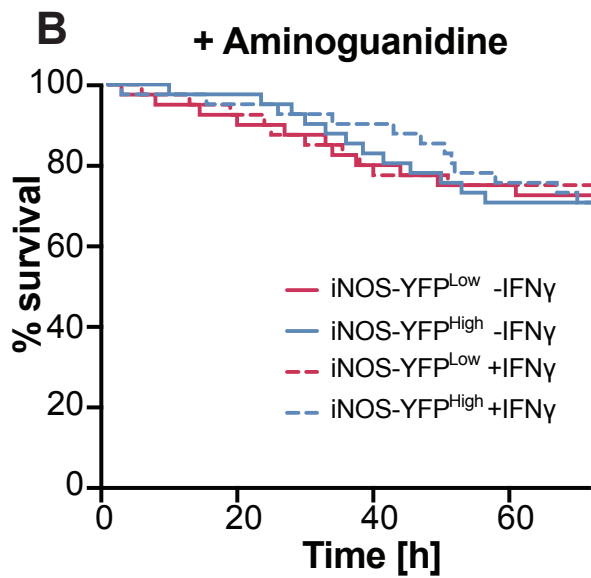

Supplement: FIG S5 [file mbio.02251-22-s0005.pdf]

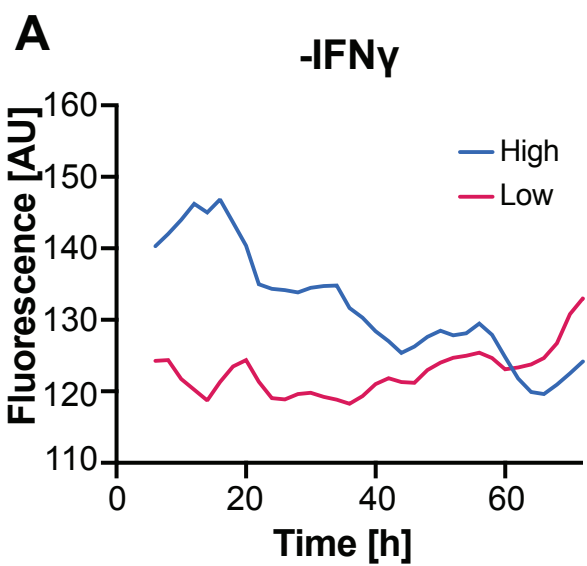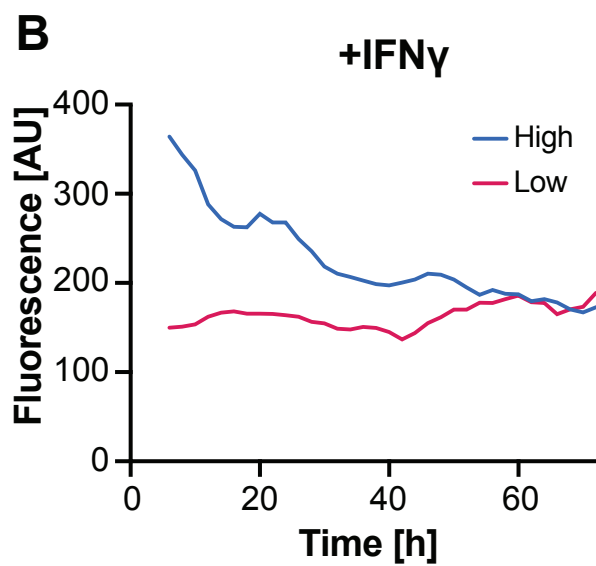

Supplement: FIG S6 [file mbio.02251-22-s0006.pdf]

**A****0-36h**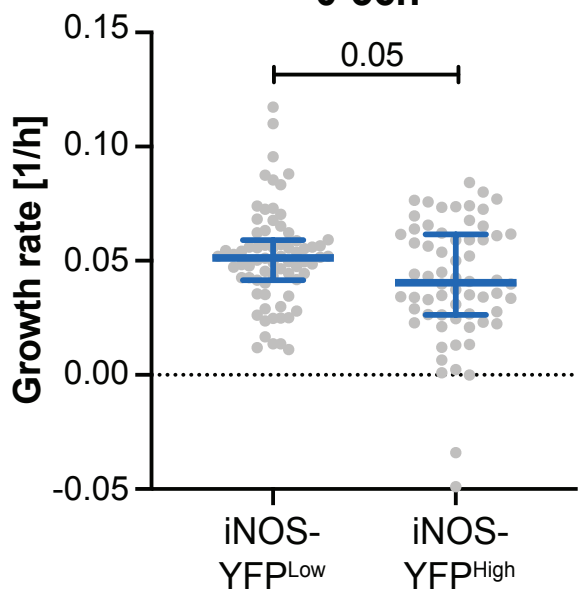**B****36-72h**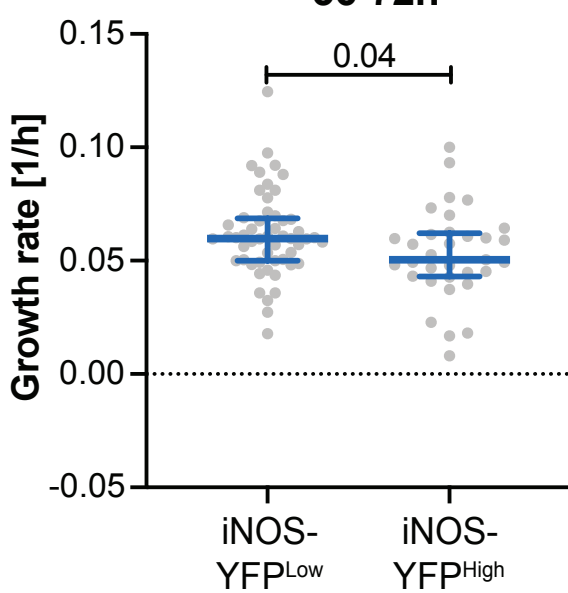

Supplement: FIG S7 [file mbio.02251-22-s0007.pdf]
