# Supplementary material for: Preexisting Heterogeneity of Inducible Nitric Oxide Synthase Expression Drives Differential Growth of Mycobacterium tuberculosis in Macrophages
Source: mBio. 2022 Sep 19;13(5):e02251-22. doi: 10.1128/mbio.02251-22 (PMC9600446; doi:10.1128/mbio.02251-22)
Supplement: TABLE S1 [file mbio.02251-22-s0008.docx]

**Primer list**

**NOS2**

| Forward Sequence | GAGACAGGGAAGTCTGAAGCAC |
| --- | --- |
| Reverse Sequence | CCAGCAGTAGTTGCTCCTCTTC |

**IRGM1**

| Forward Sequence | CATTGCCTCTGAGCAGTTCAGC |
| --- | --- |
| Reverse Sequence | CCTCTGATAGGACACTGGTGCT |

**IP10/CXCL10**

| Forward Sequence | ATCATCCCTGCGAGCCTATCCT |
| --- | --- |
| Reverse Sequence | GACCTTTTTTGGCTAAACGCTTTC |

**IFNGR1**

| Forward Sequence | CTTGAACCCTGTCGTATGCTGG |
| --- | --- |
| Reverse Sequence | TTGGTGCAGGAATCAGTCCAGG |

**IRF1**

Forward Sequence TCCAAGTCCAGCCGAGACACTA

Reverse Sequence ACTGCTGTGGTCATCAGGTAGG

**STAT1**

| Forward Sequence | GCCTCTCATTGTCACCGAAGAAC |
| --- | --- |
| Reverse Sequence | TGGCTGACGTTGGAGATCACCA |

**IFNg**

| Forward Sequence | CAGCAACAGCAAGGCGAAAAAGG |
| --- | --- |
| Reverse Sequence | TTTCCGCTTCCTGAGGCTGGAT |

**IL10**

| Forward Sequence | CGGGAAGACAATAACTGCACCC |
| --- | --- |
| Reverse Sequence | CGGTTAGCAGTATGTTGTCCAGC |

**VEGF**

| Forward Sequence | CTGCTGTAACGATGAAGCCCTG |
| --- | --- |
| Reverse Sequence | GCTGTAGGAAGCTCATCTCTCC |

**ARG1**

| Forward Sequence | CATTGGCTTGCGAGACGTAGAC |
| --- | --- |
| Reverse Sequence | GCTGAAGGTCTCTTCCATCACC |

**GAPDH**

| Forward Sequence | CATCACTGCCACCCAGAAGACTG |
| --- | --- |
| Reverse Sequence | ATGCCAGTGAGCTTCCCGTTCAG |
